# Eugenics and Parental Responsibility

**Published:** 1909-10-02

**Authors:** 


### The Hospital

A Journal of

The Medical Sciences and Hospital Administration.

New Series. No. 136, Vol. VI. [No. 1208, Vol. XLVII.] SATURDAY, OCTOBER 2, 1909.

### Eugenics and Parental Responsibility.

According to some reformers of the day, heredity, as far as regards the welfare of individuals, matters little, but environment is everything. They say, and with apparent justice which is dangerously misleading, that for a child to be born unhealthy is a relative rarity. Ergo, the prevalent excess of illness and defectiveness, whether physical, moral, or mental, must be attributed to environment, and the road to betterment is the road of social reform. This attitude has achieved so pronounced a vogue that one might be led to suppose that it was based upon incontrovertible premises. Nevertheless, this is, unfortunately, not the case. In an impressive pamphlet issuing from the Galton Laboratory for National Eugenics, Ethel M. Elderton has attempted a correlation between the physical characteristics of children, and various environmental factors in their homes. We cannot do better than quote the concluding paragraph, in which the bearing of the research is summarised. "Practically all social legislation has been based on the assumption that better environment meant race progress, whereas the link between the two is probably that a genuine race-progress will result in a better environment. The views of philanthropists, and of those who insist that the race can be substantially bettered by changed environment, appeal to our sympathies, but these reformers have yet to prove their creed. So far as our investigations have gone they show that improvement in social conditions will not compensate for a bad hereditary influence; the problem of physical and mental degeneration cannot be solved by preventing mothers from working, by closing public-houses, or by erecting model dwellings. The only way to keep a nation strong, mentally and physically, is to see that each new generation is derived chiefly from the fitter members of the generation before."

These are pregnant sentences, and, even apart from the statistical backing they have received, would appeal to thoughtful persons. It is quite easy to trace to its source the commonly-received fallacy that environment is the paramount factor in moulding a life. This fallacy is the legitimate child of the innate sense of justice which leads men to say, "All men are equal," though the inherent inequalities of mankind force themselves into view at every turn. Such things are said because the speakers revolt at the injustice implied by any other assumption. Being determined not to accept as inevitable the hardships dealt out by the unequal hand of Nature, they have managed to make themselves incapable of seeing things as they are, and by constant ingemination of their catch-word have not only themselves come to believe it true, but have even obtained for it a kind of traditional sanctity and acceptance which it by no means deserves. The truth is that the upholding of heredity as the predominant element in the construction of a life involves a degree of parental responsibility which parents are, not unnaturally, extremely loth to admit. It is far pleasanter for a man whose child is, let us say, tuberculous, or imbecile, or morally insane, to be able to say, "This was due to a chill, or to a fall in childhood, or to a careless nurse," as the case may be, than to have to arraign his conscience and admit that he ought never to have married, having himself been tuberculous or insane at some time; or, perhaps, that he ought not to have married a wife tainted with such hereditary disabilities. But, of course, acceptance of parental responsibility in its best and completest sense, which includes the responsibility of potential parentage, means the exercise of great self-control to avoid a risk which is remote, and may never be materialised. It is no wonder then that most people prefer not to admit the risk, if possible, or take it if they cannot remain blind to its existence.

Those who believe, as we do, that the conclusions of this pamphlet are just and true, that by comparison with the endowment which is directly transmitted from parents to offspring, all other considerations vanish into insignificance, may well view the future of the race with some apprehension. Everywhere we see a shrinking prolificity among those classes in which there exists some sense of the responsibility of potential parentage, while the uncontrolled instincts of the lower types among us supply a continuous accretion of individuals similarly unendowed. It is hard not to give way to pessimistic forebodings when one contemplates the logical sequel to a continuance of this process. No stock possesses so much intrinsic merit that it can permit

213609

itself indefinitely to be propagated from its least efficient elements; at least with impunity. And it is unhappily true in the present day that the less a man appreciates the responsibilities of parentage the larger is his family. It is often held up as a rebuke to the "better classes" that they marry late, and that their families are small; but to a dispassionate observer it seems no grievous sin to hesitate about bringing into the world children foredoomed, by reason of hasty and ill-considered marriages, to defective health, or children in such numbers that adequate provision cannot be made for them. If we are really concerned for the mental and physical welfare of future generations it is time we were done with beguiling ourselves that the ban of heredity can be removed by judicious environment. Nothing is more certain than that such removal is impossible, though palliation of the mischief may be achieved. The rights of the unborn do not trouble us much, but if there be one thing more than another which might be held the prescriptive right of every human being, especially in this much-vaunted civilisation of ours, it is the right of beginning life sound in body and mind. We are beginning to evince a sensible, though late, solicitude for the children that are. What is required now is an awakening of interest for the children that are to be. Let us put aside this childish disregard of the hard facts of heredity, and teach our people how true it is that figs do not grow on thistles, and that fathers and mothers are to a large extent re-incarnated in their children. If we are to maintain our greatness this truth must be hammered home, that an Englishman can commit no greater crime than to bring into the world an hereditarily enfeebled child.

